# Retraction: Noncovalently bound excited-state dimers: a perspective on current time-dependent density functional theory approaches applied to aromatic excimer models

**DOI:** 10.1039/d3ra90119j

**Published:** 2023-12-08

**Authors:** Amy C. Hancock, Lars Goerigk

**Affiliations:** a School of Chemistry, The University of Melbourne Parkville Australia lars.goerigk@unimelb.edu.au +61-3-8344-6784

## Abstract

Retraction of ‘Noncovalently bound excited-state dimers: a perspective on current time-dependent density functional theory approaches applied to aromatic excimer models’ by Amy C. Hancock *et al.*, *RSC Adv.*, 2022, **12**, 13014–13034, https://doi.org/10.1039/d2ra01703b.

We, the named authors, hereby wholly retract this *RSC Advances* article due to a series of errors. These are:

• A typo in eqn (2) that omits the minus sign in the second term of the denominator. The equation describes the extrapolation of Coupled Cluster Singles (CCS) excited-state total energies to the complete basis set (CBS) limit. Note that this does not affect any of the published CCS/CBS numbers, which were obtained correctly.

• An incorrect description of how the excited-state electron-correlation energy was determined for use in the CBS extrapolations of SCS-CC2 and CCSDR(3); note that the actual extrapolations were carried out correctly.

• A typo in the SCS-CC2/CBS(3,4) reference dissociation energy for the pyrene dimer, which impacts all benchmarking results involving this system.

• Results for all self-defined global-hybrid functionals used in our exploration of Fock exchange (FE) effects and PBE38 in the benchmarking study. This impacts the discussion of the FE study.

• Results for B2GP-PLYP-D4 due to incorrect s6 implementation in the old version (2.0) of the dftd4 program used in our work, which we were not aware of.

None of our major conclusions, insights and recommendations are affected, but numerical results are. Having consulted with an independent expert, the Royal Society of Chemistry has determined that any changes made to the paper to correct this would be major, and therefore that the best course of action is retraction and republication of the article with the correct data. The Royal Society of Chemistry is happy that the overall conclusions of the paper are not affected by these errors, and therefore that republication of the work with the correct data is appropriate. The republished article was peer reviewed and can be found at https://doi.org/10.1039/D3RA07381E.

We, the authors, brought this matter to the attention of the Royal Society of Chemistry ourselves, and are happy with the decision to retract and republish this article.

Signed: Amy C. Hancock and Lars Goerigk, 23rd November 2023

Retraction endorsed by Laura Fisher, Executive Editor, *RSC Advances*

## Supplementary Material

